# MPT 64 Antigen detection for Rapid confirmation of *M.tuberculosis *isolates

**DOI:** 10.1186/1756-0500-4-79

**Published:** 2011-03-24

**Authors:** Vijay GS Kumar, Tejashree A Urs, Rajani R Ranganath

**Affiliations:** 1 Professor & Head of Clinical Microbiology, JSS Medical College, Mysore, 570 015, India; 2 Professor of Microbiology, JSS Medical College, Mysore, 570 015, India; 3 Post graduate student, Department of Microbiology, JSS Medical College, Mysore, 570 015, India

## Abstract

**Background:**

A new rapid Immunochromatographic test kit(SD MPT64TB Ag Kit) for detection of MPT 64 Antigen in *M. tuberculosis *isolates using mouse monoclonal MPT 64 Antibody developed by SD Bioline, South Korea was evaluated for rapid identification of *M. tuberculosis *isolates. We also assessed the sensitivity, specificity and predictive values of this kit. The test kit has an excellent sensitivity, specificity, negative predictive value & positive predictive value. This rapid method is found to be a reliable, rapid and cheaper method for confirming MTB culture isolates in resource poor laboratories. Material/methods: 54 culture isolates of *M. tuberculosis *in broth & on LJ medium, 12 Non mycobacterial isolates, 10 Non tubercular (NTM) rapidly growing Mycobacteria isolated from pus & 5 smear positive sputum samples were tested for detection of MPT64 antigen using the SD Bioline immunochromatography (ICT)test kit. H37 RV strain was employed as the positive reference control.

**Findings:**

H37 RV strain showed the presence of MPT64 antigen band. Similar band was formed in all the 54 MTB isolates tested proving 100% sensitivity. MPT64 band formation was not detected in any of the other test isolates which proved the 100% specificity of the test kit. Both PPV & NPV were 100%.

**Conclusion:**

Tuberculosis is a global pandemic. Rapid identification of MTB culture isolate is very important for drug susceptibility testing. MPT 64 TB Ag detection ICT kit is a rapid, reliable method; it can be a substitute for the molecular identification methods.

## Introduction

Tuberculosis (TB) is a Global pandemic & India is the major TB endemic country. Emergence of multi drug resistance(MDR) in *M. tuberculosis*(MTB) & the TB-HIV AIDS nexus is posing serious therapeutic problems and hampering the goal of WHO in containing this dreadful disease in India. Slow growing nature of MTB along with the tedious, risky & cumbersome identification tests collectively has led to long delay in antitubercular susceptibility testing. This prolonged time needed for correct identification of MTB isolates is probably one of the factors contributing to increase of TB cases in India. Automated culture systems like Bactec 460, MBBacT (Bio Merieux, France), MGIT have significantly reduced the turn around time for culture, but do not help in differentiating MTB & MOTT. Confirmatory Identification of M. tuberculosis (MTB) is still being done by conventional biochemical methods. These tests are laborious, time consuming and require elaborate safety precautions. During this long and tedious process, an infected individual continue to spread the disease to many other susceptible individuals. The recent objective of WHO is to reduce the time for culture, identification, and drug resistance detection to as short as 2 days by employing Line probe assays. This method requires a specialized set up, trained personale & is not suitable for resource poor countries.

An alternative rapid and reliable method of Identifying MTB culture isolates was being continuously searched by the researchers. The hard work paid dividends in the form of identifying a 28kDa antigen called MPT64 which is specific for MTB and not found in BCG strains [1] Recently it has been shown that native as well as recombinant MPT64 will distinguish between MTB infection & BCG vaccinated individuals [2] This antigen detection in culture isolates has been found to be a highly specific, sensitive and rapid method of confirming MTB isolates. SD TB Ag MPT 64 Rapid (Standard Diagnostics, Seoul, South Korea) has manufactured an Immunochromatographic test (ICT) using Monoclonal Antibodies against MPT64 antigen for confirmation of MTB isolates [3,4]

In this direction we evaluated the clinical usefulness, specificity, sensitivity, positive predictive (PPV) & Negative predictive value (NPV) of the commercial Immunochromatographic test kit, SD TB Ag MPT 64 Rapid to prove the specificity of MPT 64 Ag to only MTB complex.

## Materials and methods

54 MTB confirmed isolates, one H37RV culture, 10 MOTT isolates from pus, 12 Gram pos & Gram negative bacterial isolates from sputum & Urine (Table 1) were tested for the presence of MPT64 antigen by Immunochromatographic method.

**Table 1:** Sample distribution

Nature of sample	Number	Mpt64 negatives	Total number
H37Rv	1	0	01
MTB culture isolates	54	0	54
Nontubercular Mycobacteria	10	10	10
Non mycobacterial isolates	12	12	12

**Total**	**77**	**22**	**77**

## Methods

Manufacturer's instructions were followed during the test. The entire test procedure was carried out inside a biosafety cabinet category -II (BSC-II).

3- 4 colonies from L.J. Medium isolates & H37 Rv strain were emulsified in 200 μl. of extraction buffer. The buffer was vortexed, 100 μl from this buffer was inoculated in to the sample well of the ICT cassette.100 μl of the culture positive broth (BacT/alert MP bottles) was directly inoculated into the test cassette. Similarly 3-4 culture colonies of each non mycobacterial isolates (*E. coli, K. pneumoniae, S. typhi, P. aeruginosa, Acinetobacter baumanii, S. aureus*) were emulsified in the buffer & 100 μl was inoculated in to the sample well.

5 smear positive samples after initial homogenization were further processed by adopting different methods. One sample was centrifuged at 3000 rpm for 10 mts, 100 μl of the supernatant was inoculated in to the sample well. 100 μl from each of the other two sputum samples was mixed with 200 μl of the buffer & 100 μl from this was inoculated in to the sample well, 100 μl from the other two sputum samples was directly inoculated into the sample well.

The inoculated ICT cassettes were kept at room temperature & were examined at the end of 20 minutes. Pink band in the 'C' region confirmed test validity. Additional pink band in the 'T' region was interpreted as positive for MPT64 Ag. Only pink band in the 'C' region and no band in the 'T' region were considered negative for MPT 64 antigen. No band in 'C' region was interpreted as invalid test.

## Results

The control band(C band) was seen in all the 77 samples tested validating the test. H37 Rv control showed the appearance of pink band in the test region (T band) confirming the presence of MPT 64 antigen. 53 *M. tuberculosis *isolates showed the dark pink band in the test region confirming the presence of MPT 64 Ag in these isolates. One isolate showed a faint pink band formation (54/54). The band intensity is probably related to the antigen concentration in the test sample or it could be due to any mutation in the isolate. The sensitivity of the test kit was 100%. The isolate showing faint band has been sent for genetic characterization. None of the other 22 test samples including the MOTT isolates showed the pink band formation, indicating the absence of MPT64 Ag in these isolates. (Table 2). This proved the specificity of the ICT (100%).

**Table 2:** MPT64 Test results

Nature of sample	Number tested	Mpt64 negatives	MPT64 pos
H37Rv	1	0(100%)	01(100%)
MTB culture isolates	54	00(100%)	54(100%)
Nontubercular Mycobacteria	10	10(100%)	00(100%)
Non mycobacterial isolates	12	12(100%)	00(100%)

**Total**	**77**	**22**	**55**

The study also evaluated the ability of SD TB MPT 64 Ag Rapid ICT kit for antigen detection in smear positive sputum samples, no band appeared in all the 5 sputum samples tested at the end of 20 mts, but the band appeared after overnight incubation. Probably modification of the antigen extraction method leading to increased antigen concentration is needed for this rapid test to be used for identification of MTB in clinical samples. The negative test cassettes were examined for test band appearance after overnight incubation at room temperature, none of these showed the test band.

## Discussion

Tuberculosis is an endemic disease of great antiquity. It has conquered the entire world and has not spared any race or creed. Tuberculosis (TB) is a major public health problem in India. India accounts for one-fifth of the global TB incident cases. Each year nearly 2 million people in India develop TB, of which around 0.87 million are infectious cases. It is estimated that annually around 330,000 Indians die due to TB. TB is one of the leading causes of mortality in India killing 2 persons every three minutes, nearly 1,000 every day. Early diagnosis well supported by a Mycobacteriology laboratory equipped to perform culture, identification and drug susceptibility testing of *Mycobacterium tuberculosis *from clinical specimens is vital in the management of tuberculosis patients. The automated systems have improved the speed of isolation but there is a need for rapid identification of MTB isolates. Novel technologies for rapid identification of the culture isolates and Anti tubercular drug resistant isolates has become a top priority not only in TB Research but also for diagnosis & treatment purposes.

The modern molecular methods are not economically suitable for resource poor laboratories. A cheap, rapid & reliable identification test method will be of enormous help in resource poor countries. The new rapid Immunochromatographic methods have been found to be such ideal diagnostic aid in TB control programme

The main aim of this study was to evaluate a rapid & economically feasible test which accurately identifies MTB isolates grown in culture [1]. Clinically and therapeutically differential identification of *M. tuberculosis *from mycobacteria other than *M. tuberculosis *(MOTT) is very important. Most of the Mycobacteriology laboratories identify MTB using conventional biochemical tests. These tests are not labor sensitive & need special bio safety equipments. Biological, molecular and immunological studies of MTB complex have resulted in identification of different useful antigens, some of which are specific to MTB complex. Tuberculosis MPT64 also termed as protein Rv1980c is one such antigen [5,6]. It is a protein secreted by actively growing MTB strains [7]. The MPT 64 antigen is absent in BCG strains, *M. bovis *&*M. leprae *and in other Mycobacterial species. This has been confirmed by Cloning and sequencing of MPT64 gene of H37Rv culture filtrate [8]. The ongoing MTB research studies have proved the immunogenic property of MPT64 [9,10]. MPT64 antigen and culture filtrate protein (CFP-2) antigens are restricted antigens of MTB complex and are found to be TB specific candidate antigens [11]. It has also been proved that MPT 64 antigen is found only in Viable & actively dividing cells of *M. tuberculosis *[5]. Acommercial ICT test kit (Capilia TB kit) was evaluated for Rapid Identification of 784 culture isolates by employing Accu probe -MTB as the reference method for MTB identification & comparative evaluation of the rapid kit. The sensitivity of the rapid test kit was found to be 99.2%. (381/384). An important observation in this study was no false positive results were detected. Thus the study found the specificity of the rapid ICT test as 100% & have concluded that the Capilia TB ICT test kit is an useful method for rapid & routine identification of MTB isolates[3,12]. Comparative evaluation studies of Rapid ICT test kits & conventional biochemical tests with Accu probe sequencing has proved that these ICT's have 100% specificity & a sensitivity of 97%. The Rapid ICT's are economically cheaper & the turnaround time for specifically identifying MTB is markedly reduced [4]. SD MPT 64 TB Ag rapid ICT kit with its Very high sensitivity, high specificity, no false positivity & the low tech rapidity scores over the molecular methods[1]. The sensitivity, specificity, positive predictive & negative predictive values of the SD AgMPT64 kit was found to be 97%, 100%,100% & 92% respectively [13]. No false positive or false negative results were detected in the study. ICT's detecting MPT64 antigen could be the replacement for conventional identification tests in the days to come [1]. The simplicity of the method, low cost compared to molecular methods for confirmation of MTB isolates makes the rapid ICT's an excellent choice for use in TB diagnostic laboratories. In the present study the sensitivity of SD Ag MPT 64 kit was 100%. All 54 MTB isolates showed same band similar to H37Rv culture. None of the non mycobacterial species & the non tubercular mycobacterial species tested showed Band formation for MPT64 antigen & absence of this antigen in other non tubercular isolates indicated the specificity of MPT 64 antigen for MTB only.

The positive & Negative predictive values in the study was 100%,. A lower NPV (92%) was observed in one of the study which found 100% PPV [13]. The lower NPV can only be explained as false negative result. In contrast 100% NPV (260/260) was detected in one of the study [14]. There are reports of false negativity in ICT methods among few genetically confirmed MTB strains. This is attributed to mutation occurring in the specific gene of MTB isolates [15]. One study using Capilia TB ICT kit detected false negativity in 6 MTB isolates [4]. Genomic analysis showed mutation in all 6 strains. This is a common feature encountered with both commercial kits.

There was no difference in the predictive values, sensitivity & specificity for isolates grown in liquid culture medium and on L.J. Medium. The intensity of the band was more prominent with the liquid cultures. During the study an attempt was made to evaluate the ICT for identification of MPT 64 antigen in smear positive sputum samples. Further improvement in antigen extraction method may help in employing SD MPT 64 TB Ag Rapid kit for antigen detection in clinical samples. Similar observations have been expressed by other researchers. Cost effective analytical studies of SD MPT64 TB Ag ICT and other rapid ICT's in comparison with molecular methods and culture combined with conventional biochemical tests have shown that SD MPT 64 TB Ag ICT is more economical than the other two methods. This test does not require any sophisticated equipments or specialized trained persons [16].

## Conclusion

The SD MPT 64 TB Ag Rapid ICT kit is a simple, reliable, rapid, low-tech identification kit which can be used as a diagnostic tool in resource poor diagnostic centers for accurate identification of MTB isolates. The rapidity of this test will markedly reduce the turnaround time in MTB culture & Drug susceptibility testing. Indirectly these tests contribute in a significant way in Tuberculosis control programmes. The low cost, rapidity, High specificity, Sensitivity, PPV & NPV of the MPT 64 antigen detection makes the ICT's a very useful diagnostic tool. India being the major endemic country of tuberculosis is in need of introducing the rapid diagnostic methods more than any other country in the world

## Competing interests

The authors declare that they have no competing interests.

## Authors' contributions

GSV -- Selecting the topic of the study, Identification of non tubercular mycobacterial isolates, Interpreting all the results & preparing the manuscript. TA. - Editing the manuscript, performing the test, Identification of Non mycobacterial isolates. RR - Culture of MTB isolates, conventional identification tests, prepared the extract for the test, collected all the references. All authors have read and approved the final manuscript.
